# Human Umbilical Cord Mesenchymal Stem Cells Transplantation Promotes Cutaneous Wound Healing of Severe Burned Rats

**DOI:** 10.1371/journal.pone.0088348

**Published:** 2014-02-20

**Authors:** Lingying Liu, Yonghui Yu, Yusen Hou, Jiake Chai, Hongjie Duan, Wanli Chu, Haijun Zhang, Quan Hu, Jundong Du

**Affiliations:** Department of Burn & Plastic Surgery, the First Affiliated Hospital of PLA General Hospital, Beijing, China; University of Sao Paulo - USP, Brazil

## Abstract

**Background:**

Severe burns are a common and highly lethal trauma. The key step for severe burn therapy is to promote the wound healing as early as possible, and reports indicate that mesenchymal stem cell (MSC) therapy contributes to facilitate wound healing. In this study, we investigated effect of human umbilical cord MSCs (hUC-MSCs) could on wound healing in a rat model of severe burn and its potential mechanism.

**Methods:**

Adult male Wistar rats were randomly divided into sham, burn, and burn transplanted hUC-MSCs. GFP labeled hUC-MSCs or PBS was intravenous injected into respective groups. The rate of wound closure was evaluated by Image Pro Plus. GFP-labeled hUC-MSCs were tracked by *in vivo* bioluminescence imaging (BLI), and human-specific DNA expression in wounds was detected by PCR. Inflammatory cells, neutrophils, macrophages, capillaries and collagen types I/III in wounds were evaluated by histochemical staining. Wound blood flow was evaluated by laser Doppler blood flow meter. The levels of proinflammatory and anti-inflammatory factors, VEGF, collagen types I/III in wounds were analyzed using an ELISA.

**Results:**

We found that wound healing was significantly accelerated in the hUC-MSC therapy group. The hUC-MSCs migrated into wound and remarkably decreased the quantity of infiltrated inflammatory cells and levels of IL-1, IL-6, TNF-α and increased levels of IL-10 and TSG-6 in wounds. Additionally, the neovascularization and levels of VEGF in wounds in the hUC-MSC therapy group were markedly higher than those in other control groups. The ratio of collagen types I and III in the hUC-MSC therapy group were markedly higher than that in the burn group at indicated time after transplantation.

**Conclusion:**

The study suggests that hUC-MSCs transplantation can effectively improve wound healing in severe burned rat model. Moreover, these data might provide the theoretical foundation for the further clinical application of hUC-MSC in burn areas.

## Introduction

Severe burns are one of dangerous surgical emergency. The key step for rescuing severe burn patients is to cover the wounds as early as possible [1]. However, autogenous skin sources are seriously deficient, and xenogenic skin sources have been limited after the execution of the Regulation of Human Organ Transplantation. Meanwhile, using xenogenic pig skin is usually subjected to antigen rejection reactions and infection risk. Furthermore, bioengineered skin substitutes are still in the experimental stage. Therefore, novel and effective therapies for promoting wound healing of severe burn patients are needed.

Mesenchymal stem cells (MSCs) derive from stromal which can be isolated from multiple human tissues, such as bone marrow, adipose tissue, skeletal muscle, synovium, gingiva, amniotic fluid, umbilical cord blood, and the umbilical cord [2], [3]. MSCs are involved in the regeneration of many injured tissues, such as lung, kidney and spinal cord [4], [5], [6], [7], [8]. Compared to other original MSCs, the advantages of human umbilical cord MSCs (hUC-MSCs) are short amplification time, high proliferation rate, lower immunogenicity, higher safety, abundance, and convenience [3], [9], [10]. It has been reported that hUC-MSCs promote the functional recovery of patients with radiation-induced burns [11]. However, little is known about hUC-MSCs in the treatment of severe thermal burns.

A previous study indicates that MSCs are recruited to wounds and contribute to wound repair by transdifferentiation into multiple cutaneous cell types [12]. However, recent investigations have shown that MSC paracrine signaling is the primary mechanism accounting for the beneficial effects of MSC in response to injury [13], [14]. Therefore, the aim of the present study is to evaluate the effect of hUC-MSC on wound healing in severe burns and its potential mechanisms.

## Materials and Methods

### Animal Care

All studies adhered to procedures consistent with the International Guiding Principles for Biomedical Research Involving Animals issued by the Council for the International Organizations of Medical Sciences (CIOMS.) and were approved by the Institutional Animal Care and Use Committee at the First Affiliated Hospital to PLA General Hospital. Six-week-old male Wistar rats (180–220 g) weighing were anesthetized by intraperitoneal injection of 300 mg/kg Avertin (20 mg/ml) (2,2,2-tribromoethanol, Sigma, USA) [15]. At the end of the experiment, all rats were sacrificed with an overdose of 10% chloral hydrate.

### Animal Model of Severe Burn

The 126 adult male Wistar rats were randomly divided into 3 groups (n = 42): sham, burn, and burn transplanted hUC-MSC. Each group was divided equally into seven subgroups of six rats according to the period of euthanasia at 0, 1, 2, 3, 6, 8, 11 weeks after the cells transplantation. A model of 30% TBSA and full-thickness burn was prepared; after the rats were anesthetized by intraperitoneal injection of Avertin (300 mg/kg), the dorsal hair was removed completely, first with clippers and then through the application of Veet depilatory cream. The backside of these rats were then placed in hot water (94°C) for 12 s, which caused 30% TBSA with a full-thickness burn [15]. An immediate injection of balanced salt solution (40 mg/kg) was administered to prevent shock. The back wound was then treated with 1% tincture of iodine and kept dry to prevent infection. The rats in the sham group were placed in water at 37°C for 12 s, and the other processes were the same as those applied to the burned rats. Wounds were left open and animals were sacrificed at defined time points after transplantation.

### Isolation, Culture, and Labeling of hUC-MSCs

The hUC-MSCs were isolated from 3 full-term healthy fetuses who was born via caesarean delivery (gestation age, 39–40 weeks), expanded *in vitro*, and characterized as previously described [16]. hUC-MSCs from passages 3–8 were used for all experiments. Two days before transplantation, hUC-MSCs were labeled with green fluorescent protein (GFP) using a lentiviral strategy. The positive rate of GFP-labeled hUC-MSCs was assayed by flow cytometry [17].

### Intravenous Injection of GFP-labeled hUC-MSCs

The adult male rats were randomly divided into 3 groups. The rats in the burn transplanted hUC-MSC group received a tail vein injection of 5×10^6^ GFP-labeled hUC-MSCs at day 3 after burn. The rats in the other groups received a tail vein injection of PBS at the same time. All rats in each group were clinically evaluated.

### Specimen Collection and Detection

At weeks 0, 1, 2, 3, 6, 8, and 11 after hUC-MSC transplantation, the complete wound with a 0.5-cm margin was carefully removed, rinsed in PBS. Then every wound specimen was divided into two pieces through the least healed portion. One piece of the wound was stored in liquid nitrogen for future molecular dectection or ELISA assay, and another piece was fixed in 4% paraformaldehyde for histological examination.

### Images Capture and Assay

GFP-labeled hUC-MSCs were applied to the severely burned rats to detect the migration pattern of hUC-MSCs *in vivo* using bioluminescence imaging (BLI, Caliper Life Sciences Inc., MA, USA). To evaluate the development of severe burn wounds, wound images were obtained and the healing time and healing rate of the wounds were evaluated at defined time points after transplantation. A residual wound area (<1%) was considered as complete healing of wound. Wound areas were measured photographically every week after hUC-MSCs transplantation, and the rate of wound closure was calculated as follows: Wound closure rate (%) = [(original wound area – wound area of defined time-points)/original wound area] × 100%. Quantitative measurements of the wound area were assessed using Image Pro Plus 5.1 image analysis software (Media Cybernetics, Silver Spring, MD).

### Human-specific DNA Assay

The cutaneous wounds of rats in the 3 groups at defined time points after hUC-MSCs injection were harvested, washed in PBS, and digested using proteinase K. Then, the DNA of each sample was extracted through phenol/chloroform purification and isopropanol precipitation. The presence of human-specific DNA was detected by PCR amplification of a 479-bp fragment of a highly repetitive α-satellite DNA sequence of the centromere region of human chromosome 17, with primers modified according to Becker (5′-GGG ATA ATT TCA GCT GAC TAA ACA G-3′; 5′-AAA CGT CCA CTT GCA GTT CTA G-3′) [18]. The 40 cycles consisted of denaturation for 30 s at 95°C, annealing for 30 s at 58°C and extension for 40 s at 72°C were performed. The product of PCR was detected using agarose gel.

### Histological Analyses

After fixation with 4% paraformaldehyde for 24 h at room temperature, the specimens were embedded in paraffin and sectioned in a plane perpendicular to the incision. Five-micrometer-thick sections were prepared, deparaffinized in dimethylbenzene, and rehydrated. Preparative sections were stained with H&E in accordance with standard procedures. Other sections were incubated with specific antibodies (monoclonal mouse antibodies against rat c-ANCA, ED-1, CD31, vWF, collagen type I or collagen type III; Santa Cruz Biotechnology, Santa Cruz, CA), followed by incubation with the corresponding secondary antibody and the PAP (peroxidase–anti-peroxidase) complex, and exposure using DAB (3,3′-diaminobenzidine). The inflammatory cells, c-ANCA+ and ED-1+ cells, capillaries and collagen types I and III in the wounds were counted in 5 randomly selected fields of the each slide by an experienced and independent cell scientist in a blinded manner.

### Laser Doppler Blood Flowmetry (LDF)

The microcirculation changes in the cutaneous wounds at defined time points after transplantation were evaluated using LDF (Periflux 5000; probe 404 device, Perimed, Sweden) [19]. LDF was measured according to the instructions of the product specification booklet as provided by the company. Briefly, specialized double-faced adhesive tape was used to bond the probe and rat skin/wound, and the BPU (Blood Perfusion Unit) value was read by the computer. The room temperature was maintained at 23.6°C ±1°C, and the humidity was 46%±2%.

### Enzyme-linked Immunosorbent Assay (ELISA)

The levels of IL-1, IL-6, TNF-α, IL-10, TSG-6, VEGF, collagen types I and III in the severely burned wound were determined using ELISA kits (R&D Systems, Minneapolis, MN). The wound specimens were cut into small pieces, and the extraction of collagen types I and III in wound tissues were performed using the standard method previously described [20]. Other samples were homogenized using a mortar and lysed in 500 ml of extraction buffer and centrifuged at 10,000 rpm for 10 min. The supernatants of all specimens were detected by a multidetection microplate reader using a double-antibody sandwich ELISA kit according to the manufacturer’s protocols. The concentrations of IL-1, IL-6, TNF-α, IL-10, TSG-6, VEGF, collagen types I and III were normalized to the total protein content.

### Statistical Analysis

All data are expressed as the mean ± SD ( ± s) and were analyzed using SPSS 16.0 (SPSS Inc., Chicago, IL, USA). The data were analyzed using independent Student’s t-test for comparison between 2 groups and Wilcoxon signed-rank test for densitometric data. When more than 2 groups were present, the ANOVA test (factorial design) was applied using Prism software (GraphPad Software, La Jolla, CA, USA). The differences were considered to be statistically significant at *P*<0.05.

## Results

### hUC-MSCs Injection Promoted wounds Recovery of Severe Burn

**Figure 1:**
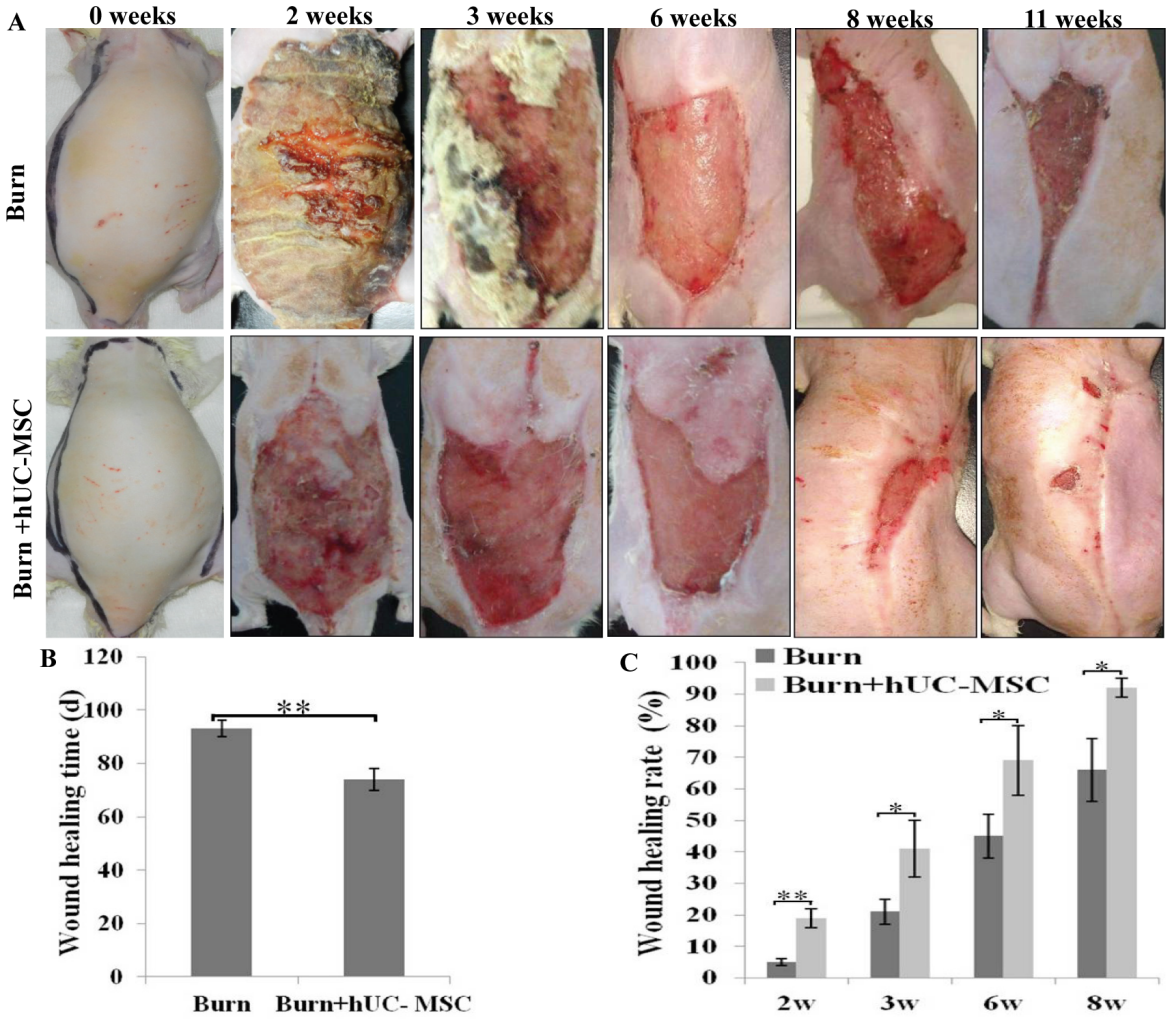
hUC-MSCs injection promoted wounds recovery of severe burn. (A) Representative images of cutaneous wounds at different time points after hUC-MSC transplantation. (B) Statistical analysis of healing time of the wounds was presented in the histogram. The healing time of wounds in the burn transplanted hUC-MSCs group was significantly shorter than that in the burn group. (C) Statistical analysis of healing rate of wounds at 2, 3, 6, and 8 weeks after transplantation was presented in the histogram. The healing rate of wounds in the burn transplanted hUC-MSC group was significantly higher than that in the burn group at same time. Values are represented as mean±SD (n = 6), asterisk (*) and double asterisk (**) stand for P<0.05 and p<0.01 compared with burn group, respectively.

The complete healing of cutaneous wounds, i.e., with good epithelization and a residual wound area of <1%, in severe burn rats was evaluated by general observation. The recovery time for the cutaneous wounds in the burn transplanted hUC-MSCs group was significantly shorter than that in the burn group. At week 2 after cell transplantation, eschars in the burn transplanted hUC-MSCs group were completely desquamated, and wounds without severe infection were clean. However, eschars in the burn group remained tightly adherent to the wounds, and purulent secretion under the eschars led to partial solution of eschars and severe infection of wounds which led to delayed healing of the wounds in severely burned rats. At week 11 after hUC-MSC transplantation, the wound epithelium of 5 of 6 rats had recovered in the hUC-MSC group, whereas all 6 rats in the burn group displayed a lack of healing at the same time (Fig. 1A). The healing time of wounds in the burn transplanted hUC-MSC and burn groups was 74±4 d and 93±3 days, respectively. The healing time for the wound in the burn transplanted hUC-MSC group was significantly shorter than that in the burn group (**P<0.01; Fig. 1B), and the healing rate of wounds in the burn transplanted hUC-MSC group was significantly higher than that in the burn group at weeks 2, 3, 6, and 8 after transplantation (*P<0.05, **P<0.01; Fig. 1C).

### Labeling and Migration of hUC-MSCs

**Figure 2:**
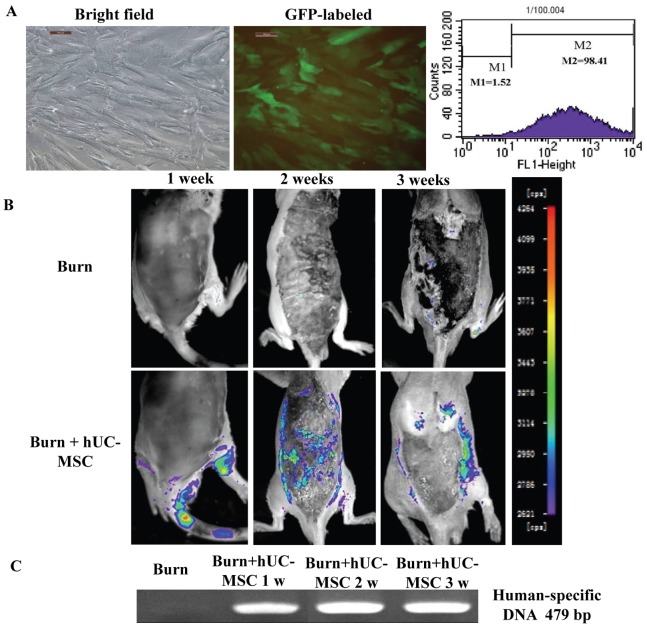
hUC-MSCs were cultured, labeled, and tracked in vivo in rats with severe burns. (A) P3 hUC-MSCs were observed by bright-field microscopy using an inverted microscope. GFP-labeled hUC-MSCs were observed by inverted fluorescence microscopy, and positive rates of GFP-labeled cells were evaluated by flow cytome try. The magnification was 100 times. (B) GFP-labeled hUC-MSCs migrated into cutaneous wounds of rats with severe burns at different time points after cell transplantation. (C) The human-specific DNA in cutaneous wounds of rats with severe burns was assayed using RT-PCR.

The hUC-MSCs from passages 3–8 had a uniform spindle shape and were in their logarithmic multiplication cycle. The hUC-MSCs were labeled with green fluorescent protein (GFP) using a lentiviral vector strategy. Our pilot data demonstrate that this strategy is highly effective in producing infectious viral particles expressing GFP (95.2%±3.5%) in grafted hUC-MSCs (Fig. 2A).

To assess the dynamic trafficking and homing of hUC-MSCs in response to severe burn wounds *in vivo*, 5×10^6^ GFP-labeled hUC-MSCs were transplanted into rats with severe burns, and sequential BLI imaging was performed from 1 to 3 weeks after hUC-MSC transplantation. On week 1 after transplantation, we observed that hUC-MSCs migrated into the severe burn wounds, and these GFP-labeled hUC-MSCs mainly concentrated in the wound edge and wound base on weeks 2 and 3 after hUC-MSCs transplantation. (Fig. 2B).

There was no PCR product of human-specific DNA in the wounds of severe burn group, but the PCR signals of human-specific DNA were presented in the wounds of severe burn transplanted hUC-MSC group at weeks 1, 2, 3. (Fig. 2C).

### Anti-inflammatory Effect of hUC-MSCs in the Wound

**Figure 3:**
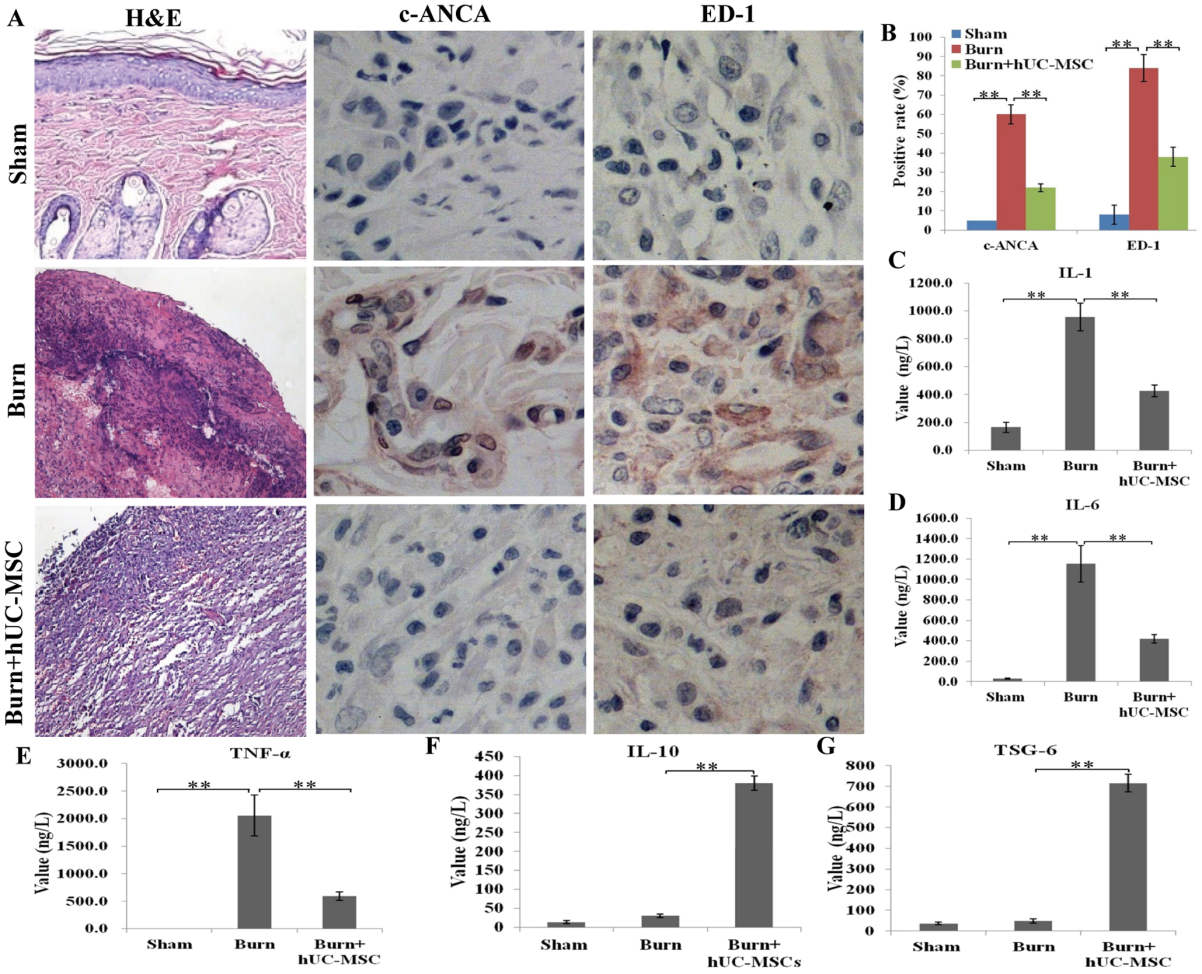
Anti-inflammatory effect of hUC-MSCs on cutaneous wounds of rats with severe burns was detected (A) The inflammatory cell infiltration in wounds was assessed by H&E staining. Positive staining for neutrophils (c-ANCA) and macrophages (ED-1) was examined by immunohistochemistry. (B) A quantitative analysis of positive staining for neutrophils (c-ANCA) and macrophages (ED-1) is shown in the corresponding histogram. (C, D, E) The proinflammatory cytokines IL-1, IL-6, and TNF-α in wounds were assessed using an ELISA kit. IL-1, IL-6, and TNF-α levels in the burn transplanted hUC-MSC group were significantly lower than those in the burn group, and their levels in the burn group were significantly higher than those in the sham group. (F, G) The anti-inflammatory cytokines IL-10 and TSG-6 in wounds were assessed using an ELISA kit. IL-10 and TSG-6 levels in the burn transplanted hUC-MSC group were significantly higher than other control groups. Values are represented as mean±SD (n = 6), double asterisk (**) stands for p<0.01.

H&E staining of the cutaneous wound sections revealed that the primary infiltrating cells in the severely burned wound were inflammatory cells. The total number of inflammatory cells in the burn transplanted hUC-MSCs group was markedly lower than that in the burn group and number of inflammatory cells in the burn group higher than that in sham group, and an abscess was observed in the burn group. The number of infiltrating neutrophils (c-ANCA^+^) and macrophages (ED-1^+^) in the cutaneous wounds in the burn transplanted hUC-MSCs group was significantly lower than that in the burn group and positive rates of c-ANCA and ED-1 in the burn group were markedly higher than that in sham group (Fig. 3A). The results of the quantitative analysis are presented in the corresponding histogram (Fig. 3B).

Furthermore, we assessed the levels of the main proinflammatory factors (IL-1, IL-6, and TNF-α) and anti-inflammatory cytokines (IL-10 and TSG-6) in the cutaneous wounds in the 3 groups on weeks 2 after transplantation. We found that the levels of IL-1, IL-6, and TNF-α in the burn transplanted hUC-MSCs group were significantly lower than those in the burn group, and the levels of these cytokines in the burn group were significantly higher than those in the sham group (Figs. 3C, 3D, 3E). However, the levels of anti-inflammtory cytokines, such as IL-10 and TSG-6, were significantly higher than other two groups (Figs. 3F, 3G).

### Effects of hUC-MSCs on Wound Neovascularization

**Figure 4:**
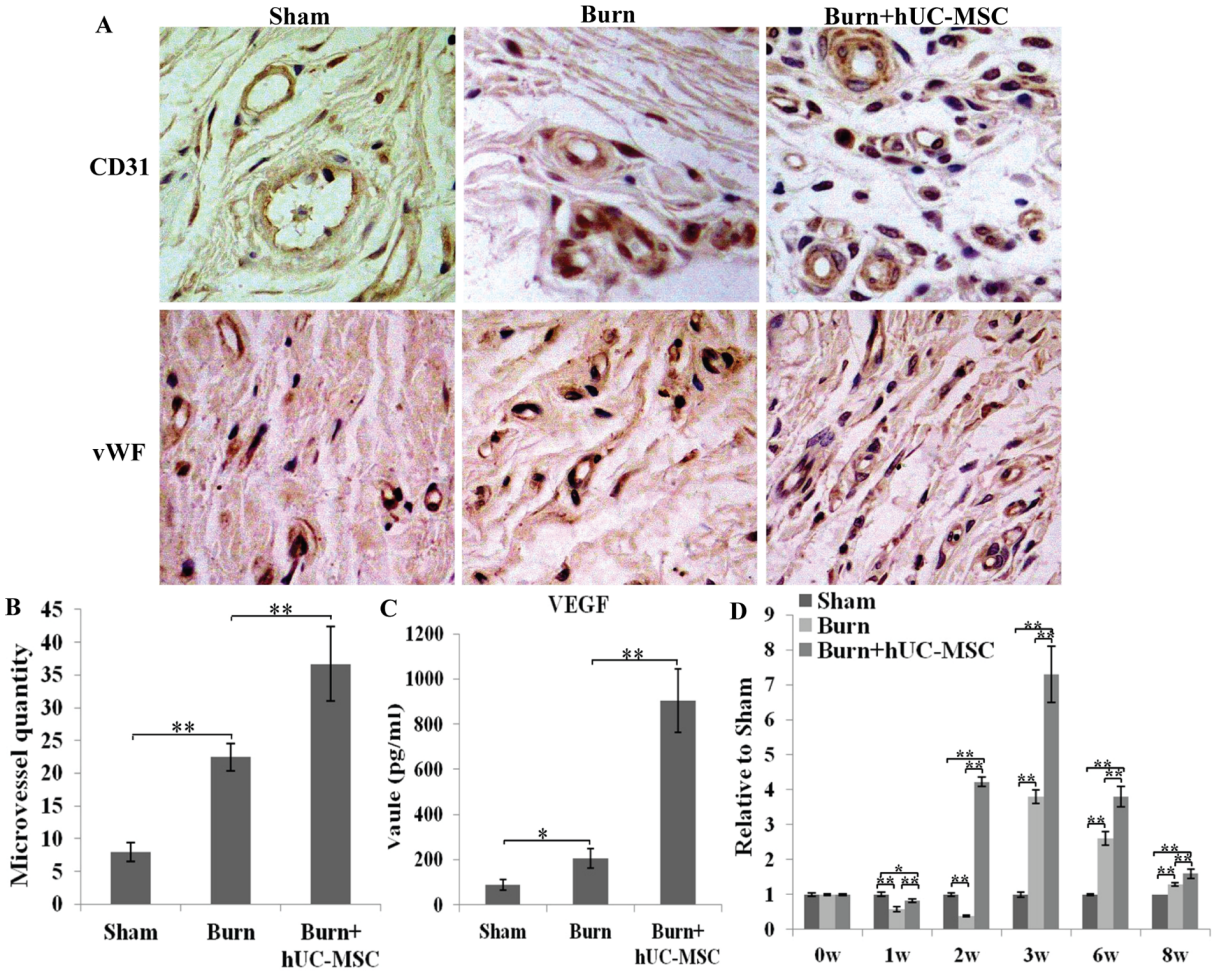
Effects of hUC-MSCs on wound neovascularization at week 3 after cell transplantation. (A) The wound samples in sham, burn, and burn transplanted hUC-MSC groups were stained for the vascular endothelial cell markers CD31 and vWF using immunohistochemistry. (B) A quantitative analysis of microvessels is shown in the corresponding histogram. (C) The level of VEGF in wounds was evaluated by ELISA kit. The VEGF level in the burn transplanted hUC-MSC group was significantly higher than that in other control groups. (D) Cutaneous wound microcirculation was evaluated using a laser Doppler blood flow meter. The cutaneous wound microcirculation in the burn transplanted hUC-MSC group was markedly higher than that in the burn group at weeks 1, 2, 3, 6 and 8 after transplantation. Values are represented as mean±SD (n = 6), asterisk (*) and double asterisk (**) stand for P<0.05 and p<0.01 respectively.

At week 3 after hUC-MSC transplantation, wound neovascularization occurred in both the burn and burn transplanted hUC-MSC groups. The neovascularization was quantified by counting the microvessels. Compared to the burn group, the burn transplanted hUC-MSC group contained more microvessels (Fig. 4A). The results of the quantitative analysis are presented in the corresponding histogram (Fig. 4B).

We further investigated the level of VEGF in the cutaneous wounds to determine the possible effect of hUC-MSCs on wound angiogenesis. The VEGF levels in the burn transplanted hUC-MSC groups were significantly higher than those in other control groups (Fig. 4C), which indicates that hUC-MSCs resulted in an increase in wound angiogenesis.

Cutaneous wound microcirculation was evaluated using a laser Doppler blood flow meter (LDF). Cutaneous wound microcirculation in the burn transplanted hUC-MSC group was markedly higher than that in the burn group at 1, 2, 3, 6, and 8 weeks after transplantation (Fig. 4D).

All results suggest that hUC-MSCs promoted neovascularization of severe burn wounds.

### Modification Collagen Types I and III Accumulation in the Wound

**Figure 5:**
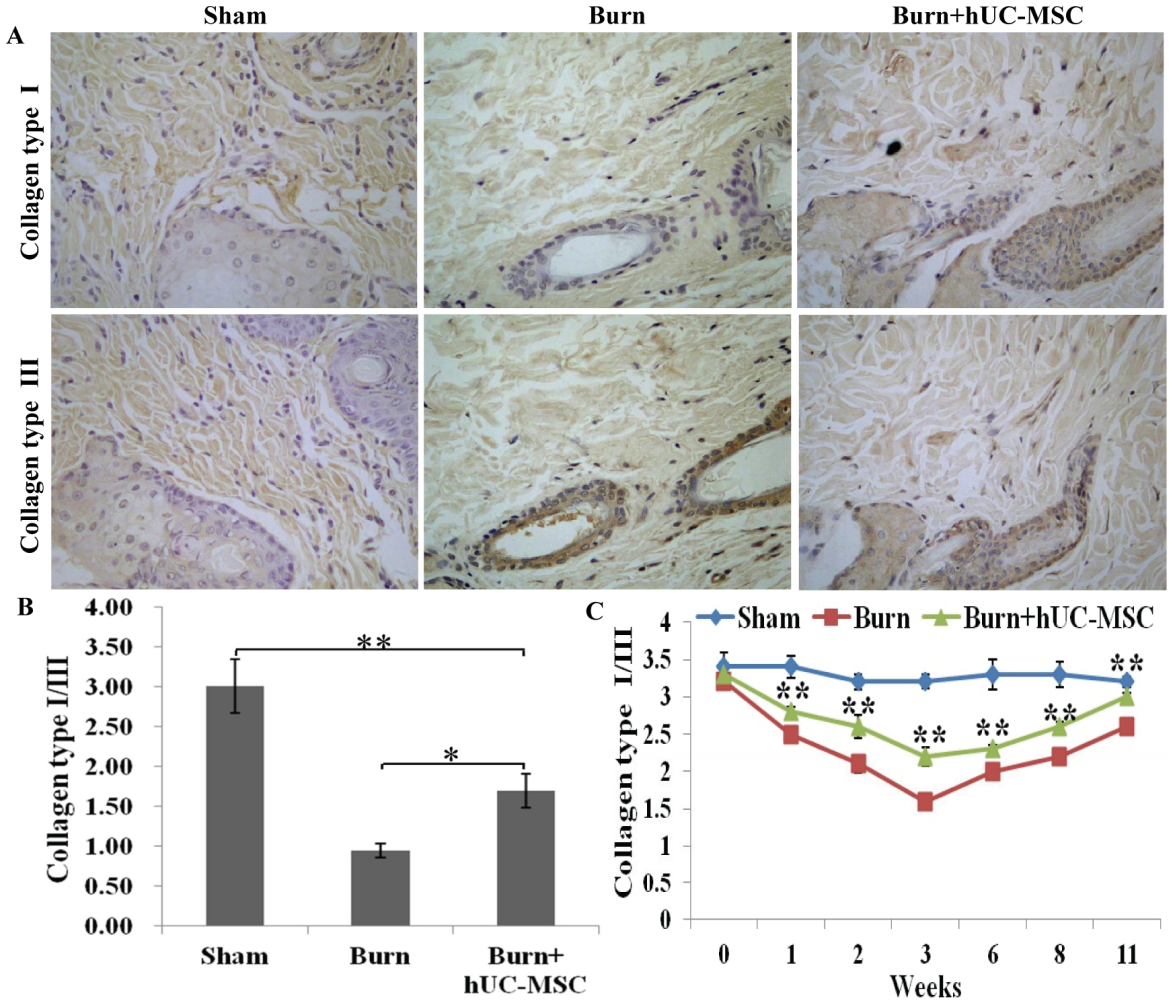
Modification collagen types I and III accumulation in the wound. (A) Treatment of severely burned wounds with hUC-MSCs modified accumulation of collagen types I and III at week 3 after transplantation. (B) A quantitative analysis of ratio of collagen types I and III is shown in the corresponding histogram. (C) The levels of collagen types I and III in wounds at different time points after transplantation were assessed using an ELISA kit. Compared with wounds in severely burned group, hUC-MSCs significantly upregulated the ratio of collagen types I and III in the wound at weeks 1, 2, 3, 6, 8 and 11 after transplantation. Values are represented as mean±SD (n = 6), asterisk (*) and double asterisk (**) stand for P<0.05 and p<0.01 respectively.

The collagen types I and III are the main collagen types of healthy skin and the ratio of collagen types I and III determined progress of wound repair. Treatment of severely burned wounds with hUC-MSCs modified accumulation of collagen types I and III at week 3 after transplantation (Fig. 5A). The results of the quantitative analysis are presented in the corresponding histogram (Fig. 5B). The elisa result also showed similar result. Compared with wounds in severely burned group, the ratio of collagen types I and III in the burn transplanted hUC-MSCs group was remarkably upregulated at weeks 1, 2, 3, 6, 8 and 11 after transplantation (Fig. 5C).

## Discussion

Prompt and appropriate treatment of severe burns is necessary, especially in the acute phase. Recently, MSCs have become a promising approach for the treatment of radiation-induced burns [1]. Considering the therapeutic efficacy of MSCs for radiation-induced burns, there is great interest in the potential use of MSCs for treating thermal burns and severe burns. In this study, we found that a large number of GFP-labeled hUC-MSCs migrated into the wound through blood circulation. hUC-MSCs-treated wounds showed more rapid wound closure, presumably because hUC-MSCs decreased inflammatory response, promoted angiogenesis and modified collagen types accumulation in the locally burned skin at different time points after transplantation by paracrine cytokines.

In the present study, hUC-MSCs migrated into wound and located in the wound edge and wound base. But the mechanism responsible for hUC-MSC homing to wounds is not well understood, it likely involves the complex interplay of adhesion molecules, chemokines and extracellular matrix proteases [21]. One study has demonstrated that intradermal injection of the chemokine SLC/CCL21 at the wound edge increased recruitment of intravenously injected MSCs and significantly accelerated wound closure [22].

Some studies confirmed that the wound healing process can be disturbed by infection, necrosis and excess levels of inflammatory factors. Even a continuous state of inflammation in the wound created a cascade that perpetuated a nonhealing state [23], [24], [25]. Our results showed that hUC-MSCs markedly attenuated the inflammation of locally burned skin by decreasing neutrophil and macrophage infiltration as well as proinflammatory cytokine production. Meanwhile, hUC-MSCs administration clearly increased the production of the anti-inflammatory cytokines IL-10 and TNF-α stimulated gene/protein 6 (TSG-6) which were main players in the anti-inflammatory cytokine profile of hUC-MSCs.

The blood supply is the key to wound healing. Several studies have demonstrated that MSC-secreted paracrine some nutrition factors such as VEGF, basic fibroblast growth factor (FGF2) and hepatocyte growth factor (HGF) promoted neovascularization of injured tissues [26], [27]. Several studies also revealed the capacity of MSCs to improve tissue vascularity by promoting endothelial cell sprouting through soluble factor secretion [28]. Our study also found that hUC-MSCs increased the level of VEGF in severe burn wounds and promote wound angiogenesis. Furthermore, we speculated that hUC-MSCs accelerated the severely burned wound healing by paracrine VEGF to increase wound angiogenesis.

Collagen as a structurally and functionally pivotal molecule, which builds a scaffold in the connective tissue, is also involved in every stage of wound healing [29]. Collagen types I and III are the main collagen types of healthy skin. Furthermore, the ratio of collagen types I and III in wounds being predominantly determined wound healing process [29]. Our results showed that hUC-MSCs can modify collagen types I and III accumulation and upregulated the ratio of collagen types I and III in the severely burned wound. A previous study also showed that MSCs promoted wound repair through secretion of collagen type I and alteration of gene expression in dermal fibroblasts [30].

hUC-MSC transplantation accelerated the wound closure of severe burns by encouraging the migration of hUC-MSCs, modulating the inflammatory environment, promoting the formation of a well-vascularized granulation matrix and collagen scaffold. These data may thus provide a theoretical foundation for further clinical application of hUC-MSCs in severe burn patients.
